# Insights into the Mechanisms of Immune‐Checkpoint Inhibitors Gained from Spatiotemporal Dynamics of the Tumor Microenvironment

**DOI:** 10.1002/advs.202508692

**Published:** 2025-08-28

**Authors:** Yuanyuan Zhang, Zhihua Liu

**Affiliations:** ^1^ State Key Laboratory of Molecular Oncology National Cancer Center/National Clinical Research Center for Cancer/Cancer Hospital Chinese Academy of Medical Sciences and Peking Union Medical College Beijing 100021 China

**Keywords:** immune checkpoint inhibitors, tumor microenvironment, spatiotemporal dynamics, spatial architecture, immunotherapy response

## Abstract

Immunotherapy, particularly immune checkpoint inhibitors (ICIs), has revolutionized cancer treatment, yet durable responses are achieved in only a subset of patients. The tumor microenvironment (TME) plays a key role in cancer progression and immune modulation, critically influencing the efficacy of ICIs. Recent advances in single‐cell technologies have enabled high‐resolution profiling of the TME, particularly tumor‐infiltrating immune cells, across diverse cancer types. However, our understanding of how immune cells shape ICI responses and how they are dynamically altered during treatment remains incomplete. In this review, we summarize recent progress in characterizing TME features associated with ICI responsiveness, highlighting key immune cell subsets involved in ICI therapy and emphasizing their phenotypic plasticity and functional adaptability following ICIs. Additionally, we outline the spatial architecture of the TME in terms of its effects on immune cell behavior and interactions, and discuss the critical role of stromal components and the microbiota in modulating the immune landscape and influencing ICI responsiveness. By integrating these insights, we aim to deepen our understanding of the cellular and molecular mechanisms underlying ICI responses, elucidate determinants of therapeutic sensitivity and resistance, and inform the development of more effective immunotherapeutic strategies.

## Graphical abstract

This review summarizes recent advances in the spatiotemporal dynamics of the tumor microenvironment (TME). It highlights the roles of diverse immune and stromal cell populations, spatial architecture of the TME, as well as the gut and tumor microbiota in influencing responses to immune checkpoint inhibitors (ICIs), by integrating insights from single‐cell and spatial transcriptomics, in order to inform biomarker discovery and improved immunotherapy strategies.

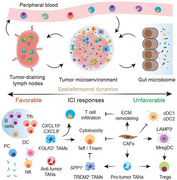

## 1 Introduction

Immune checkpoint inhibitors (ICIs) represent a groundbreaking advancement in cancer treatment. By targeting and blocking specific proteins on immune cells or cancer cells, ICIs overcome the mechanisms that cancer cells use to evade immune detection, reactivating the immune system to destroy cancer.^[^ ^1^, ^2^, ^3^ ^]^ Notable examples of ICIs include agents that target PD‐1, PD‐L1, and CTLA‐4.^[^ ^4^, ^5^, ^6^ ^]^ These therapies have shown remarkable success in treating a range of cancers,^[^ ^7^, ^8^, ^9^, ^10^ ^]^ offering new hope to patients with previously limited treatment options. However, despite their success, ICIs face several challenges in clinical practice, with one of the most significant being the variability in patient responses. Although ICIs have proven effective in many cases, a substantial proportion of patients do not experience durable or meaningful benefits.^[^ ^11^, ^12^ ^]^ This highlights the urgent need for innovative strategies to enhance ICI efficacy, as well as the development of more reliable biomarkers and predictive models.

The tumor microenvironment (TME) plays a pivotal and multifaceted role in modulating immune responses and shaping the effectiveness of ICIs.^[^ ^1^, ^13^ ^]^ A variety of factors within the TME, including cellular and non‐cellular components, interact to either promote or hinder immune responses, thereby affecting outcomes of ICI therapy.^[^ ^14^, ^15^ ^]^ These interactions are highly dynamic, with immune cells often undergoing functional reprogramming in response to both tumor‐associated signals and therapeutic interventions. For instance, T cells within the TME may shift from effector phenotypes to exhausted or dysfunctional states,^[^ ^16^, ^17^ ^]^ undermining the efficacy of anti‐tumor immune responses. In addition to immune cells, stromal components, particularly cancer‐associated fibroblasts (CAFs), play a critical role in shaping the TME by secreting extracellular matrix proteins and growth factors that can suppress immune infiltration and impair immune cell activation.^[^ ^18^, ^19^, ^20^ ^]^ Moreover, the spatial organization of these cellular components within the TME is crucial for orchestrating effective anti‐tumor immune responses and modulating ICI efficacies.^[^ ^21^, ^22^, ^23^, ^24^, ^25^ ^]^ The tumor vasculature, often abnormal in structure and excessively permeable, further complicates the interplay between the TME and ICIs.^[^ ^26^, ^27^ ^]^

Single‐cell technologies have emerged as powerful tools in cancer research, particularly for dissecting the cellular and molecular complexities of the TME.^[^ ^28^, ^29^, ^30^, ^31^ ^]^ Although the fundamental characteristics of TME components are increasingly defined, their specific roles in shaping responses to ICIs, as well as their dynamic alterations during and after treatment, remain incompletely understood. A deeper understanding of the cellular interactions and molecular mechanisms that drive ICI responsiveness is essential for improving the prediction of clinical outcomes and guiding the development of more effective, personalized immunotherapies.

In this review, we summarize recent advances in understanding the mechanisms underlying ICI therapies, with particular emphasis on studies leveraging single‐cell sequencing technologies. We highlight T cell subsets associated with ICI responsiveness, focusing on those with predictive potential and those exhibiting dynamic adaptations to ICI treatment within both the TME and peripheral tissues. In addition, we discuss the roles of other cellular components within the TME, underscoring their complex interactions and collective influence on therapeutic outcomes. We further examine the spatial organization of the TME and discuss how spatially defined features correlate with anti‐tumor immune responses and the efficacy of ICI therapies. Finally, we highlight additional factors influencing ICI responses, particularly the role of the microbiota in shaping the immune landscape and modulating treatment outcomes. By this review, we aim to advance our understanding of the cellular and molecular foundations of ICI immunotherapies and to facilitate the development of more effective and personalized cancer treatment strategies.

## 2 Genomic and Transcriptomic Features Linked to ICI Responses

Identifying reliable biomarkers to predict the efficacy of ICIs and accurately stratifying patients are critical for advancing personalized treatment and optimizing the therapeutic potential of ICIs. Clinically, PD‐L1 protein expression has become the most widely validated and accepted biomarker for guiding ICI therapies.^[^ ^32^, ^33^, ^34^ ^]^ However, its clinical application faces several challenges, including inter‐assay variability, the absence of universally agreed‐upon cut‐off points, and the need for different assays tailored to specific ICIs,^[^ ^35^, ^36^ ^]^ underscoring the critical need for standardized, consistent testing methodologies.

Genomic and transcriptomic features have been extensively explored, revealing several key factors that influence treatment outcomes. Tumor mutational burden (TMB) has been strongly associated with ICI responses.^[^ ^37^, ^38^, ^39^, ^40^, ^41^, ^42^ ^]^ Tumors with high TMB are more likely to produce neoantigens that can enhance the tumor immunogenicity and lead to immune surveillance, thereby improving ICI efficacy.^[^ ^40^, ^41^, ^43^ ^]^ Another related biomarker is neoantigen load, as a higher neoantigen load increases the tumor's visibility to the immune system, facilitating T‐cell‐mediated anti‐tumor responses.^[^ ^44^, ^45^, ^46^, ^47^ ^]^ Increased neoantigen load has been consistently associated with more durable and favorable responses to ICIs.^[^ ^38^, ^40^, ^44^, ^46^ ^]^ Although TMB is a promising biomarker, it alone is insufficient to predict ICI responses. Tumor‐specific factors, such as the immune microenvironment, immune evasion mechanisms, tumor aneuploidy, and the presence of specific mutations (e.g., BRCA1/2, PTEN, PBRM1, or JAK1), all contribute to ICI treatment outcomes,^[^ ^48^, ^49^, ^50^, ^51^, ^52^, ^53^, ^54^, ^55^, ^56^, ^57^, ^58^, ^59^ ^]^ highlighting the necessity for a more comprehensive analysis of the TME.

Regarding the evolution of tumor genomic and transcriptomic features during ICI therapies, Riaz et al. observed reductions in the TMB and neoantigen load, along with notable gene expression changes and shifts in the T cell receptor (TCR) repertoire in melanoma patients responsive to anti‐PD‐1 therapy.^[^ ^60^ ^]^ In contrast, Anagnostou et al. investigated the neoantigen landscape in ICI‐resistant non‐small cell lung cancer (NSCLC) tumors and found that acquired resistance is associated with the loss of mutations encoding tumor‐specific neoantigens, either through subclone elimination or chromosomal loss of truncal alterations.^[^ ^61^ ^]^ Notably, Łuksza et al. proposed a tumor fitness model based on immune interactions with neoantigens, identifying two key factors that determine neoantigen fitness, including the likelihood of neoantigen presentation by the major histocompatibility complex (MHC) and the subsequent recognition by T cells.^[^ ^62^ ^]^ Collectively, these studies underscore the critical role of tumor‐antigen and T cell interactions in modulating responses to ICI therapies.

## 3 Tumor‐Infiltrating T Cells and Their Implications in ICI Therapies

### 3.1 Dysfunctional States of TILs in the TME

Checkpoint therapies are designed to target T cells,^[^ ^3^, ^63^ ^]^ and T cells play a pivotal role in the effectiveness of ICIs.^[^ ^3^, ^64^, ^65^, ^66^ ^]^ Tumor‐infiltrating lymphocytes (TILs) have emerged as an early and reliable indicator of ICI efficacy.^[^ ^67^, ^68^, ^69^ ^]^ TILs, particularly CD8^+^ cytotoxic T cells, are commonly associated with a more inflamed TME and improved therapeutic outcomes. However, TILs often fail to eliminate cancer cells, as they progressively become dysfunctional or exhausted states, characterized by elevated expression of checkpoint molecules such as PD‐1, CTLA‐4, LAG‐3, TIM‐3, and TIGIT, among others.^[^ ^11^, ^17^, ^70^, ^71^, ^72^ ^]^ This phenomenon mirrors the T cell exhaustion observed in mouse models of chronic antigen exposure, such as those induced by lymphocytic choriomeningitis virus (LCMV).^[^ ^73^, ^74^, ^75^ ^]^

However, the dysfunctional states observed in human TILs differ from those seen in chronic viral infections in mouse models. Unlike exhausted T (Tex) cells in models like LCMV, which lose effector functions and eventually undergo apoptosis, dysfunctional TILs in human melanoma have been shown to consist of a highly proliferative and clonally expanded population, which exhibits a continuous spectrum of differentiation within the TME.^[^ ^76^ ^]^ Moreover, a subset of PD‐1‐bright, tumor‐reactive lymphocytes has been identified, which, while partially resembling Tex cells from chronic infections, strongly predicts responses to PD‐1 blockade and patient survival in NSCLC.^[^ ^77^ ^]^ These findings underscore the complexity and unique characteristics of dysfunctional TILs in human cancers.

Dysfunctional or exhausted T cells have been shown to exhibit distinct and stable epigenetic profiles, indicating that these cells are largely irreversible and resistant to reprogramming.^[^ ^78^, ^79^, ^80^ ^]^ Studies in mouse tumor models have reported two distinct chromatin states in T cells, a plastic dysfunctional state that can be rescued, and a fixed dysfunctional state that resists reprogramming.^[^ ^81^ ^]^ These findings underscore a critical challenge in reversing T cell exhaustion and emphasize the urgent need for systematic investigations into the mechanisms underlying ICI therapies.

### 3.2 Features of Tumor‐Reactive T Cells and Their Association with ICI Therapies

Among the diverse cellular components in the TME, tumor‐reactive T cells play a central role in ICI responses. However, distinguishing these cells within the complex TME remains a key challenge. Simoni et al. found that “bystander” CD8^+^ T cells are abundant and phenotypically distinct in human tumors, proposing CD39 as a potential marker for these non‐reactive populations.^[^ ^82^ ^]^ In contrast, Gros et al. identified PD‐1 expression on CD8⁺ TILs as a reliable indicator of clonally expanded, tumor‐reactive T cells in melanoma.^[^ ^83^ ^]^ Further insights into tumor‐reactive T cell phenotypes have emerged from studies of mutation‐associated neoantigen (MANA)‐specific T cells. Caushi et al. characterized the transcriptional profiles of MANA‐specific TILs in NSCLC patients treated with anti‐PD‐1, revealing a tissue‐resident memory T (Trm)‐like phenotype with notably low *IL7R* expression.^[^ ^84^ ^]^ Likewise, Anadon et al. reported that tumor‐recognizing TILs in ovarian cancer are predominantly Trm cells, which exhibit clonal enrichment and effector activity.^[^ ^85^ ^]^ However, Lowery et al. identified distinct signatures of neoantigen‐reactive CD8^+^ and CD4^+^ TILs, which show tumor‐specific expansion but exhibit dysfunctional phenotypes, differing from blood‐derived bystanders and regulatory TILs.^[^ ^86^ ^]^ Similarly, Oliveira et al. demonstrated that melanoma‐reactive T cells primarily exhibited an exhausted phenotype, with limited memory differentiation properties.^[^ ^87^ ^]^ Zheng et al. reported similar findings in bile duct and pancreatic cancers, where both neoantigen‐reactive CD8^+^ T cells, co‐expressing *CXCL13* and *GZMA*, and neoantigen‐reactive CD4^+^ T cells, overexpressing *HOPX* or *ADGRG1* and lacking *IL7R* expression, display an exhausted phenotype.^[^ ^88^ ^]^ These studies suggest that tumor‐reactive T cells are frequently associated with tissue‐resident or dysfunctional/exhausted phenotypes, underscoring the prevalence of Tex and Trm‐like populations. A key feature of these cells is the absence of memory‐associated IL7R expression, which may reflect their terminally differentiated state and impaired long‐term immune protection.

**Figure 1:**
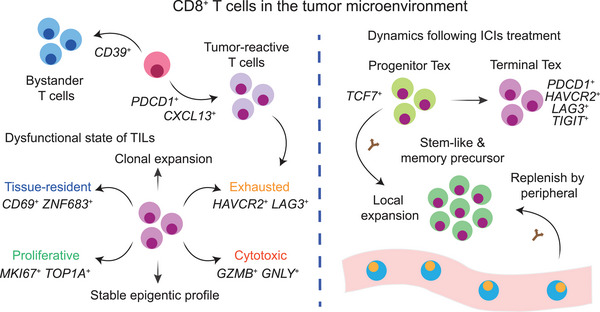
Characteristics of CD8^+^ T cells and tumor‐reactive T cells in the TME. TILs can be categorized into bystander T cells, marked by *CD39*, and tumor‐reactive T cells, marked by *CXCL13*. Tumor‐reactive T cells exhibit dysfunctional or exhausted states, along with tissue‐resident, cytotoxic, and proliferative features. They also show clonal expansion and display a specific and stable epigenetic state. Tumor‐reactive T cells can be further classified into two distinct subsets: progenitor Tex, characterized by *TCF7* expression, which exhibit stem‐like and memory precursor properties, and terminal Tex, defined by classic Tex markers. Following ICI treatment, progenitor Tex cells undergo clonal expansion, while terminal Tex cells do not. Expanded T cells after ICI therapies could also be replenished by peripheral T cell infiltration.

Identification of reliable biomarkers for tumor‐reactive T cells represents a critical step toward optimizing ICI therapies and improving patient outcomes. Hanada et al. proposed a phenotypic signature for detecting neoantigen‐reactive T cells in lung cancer, by integrating clonotype frequency, CD39 protein expression, and *CXCL13* mRNA levels through advanced multi‐omics approaches including TCR sequencing and CITE‐seq (cellular indexing of transcriptomes and epitopes by sequencing).^[^ ^89^ ^]^ Supporting these findings, Liu et al. demonstrated that *CXCL13* ^+^ tumor‐reactive T cells are linked to enhanced ICI responses across multiple cancer types, establishing *CXCL13* as a promising predictive biomarker.^[^ ^90^ ^]^ Furthermore, Litchfield et al. performed a meta‐analysis and identified both *CXCL13* and *CCR5* as key determinants of T cell responsiveness to ICIs.^[^ ^91^ ^]^ Together, these studies establish PD‐1 (PDCD1) and CXCL13 as core biomarkers of tumor‐reactive T cells (**Figure** **1**), offering clinically actionable tools for patient stratification and ICI response prediction in oncology.

Consistently, tissue‐resident features have emerged as critical determinants of both clinical outcomes and ICI efficacy. Savas et al. identified a subset of Trm cells that highly expresses immune checkpoint molecules and effector proteins and is associated with improved prognosis in breast cancer.^[^ ^92^ ^]^ Similarly, Anadon et al. showed that only Trm cells exhibit clonal enrichment and effector activity in ovarian cancer, with intra‐epithelial TCF1^low^ Trm predicting patient outcomes.^[^ ^85^ ^]^ Krishna et al. found that CD8^+^ Trm cells characterized by *CD69* and *ZNF683* are enriched in ICI responders with clear cell renal cell carcinoma (ccRCC).^[^ ^93^ ^]^ Likewise, Oliveira et al. showed that preexisting Trm cells, marked by high cytotoxic potential and *ZNF683* expression, are associated with favorable responses to neoadjuvant anti‐PD‐1 therapy in head and neck cancer.^[^ ^94^ ^]^ Additionally, Virassamy et al. reported that ICIs notably enhance the cytotoxicity of Trm‐like subsets, which confer local protection against tumor rechallenge and correlate with improved outcomes in ICI‐treated patients with triple‐negative breast cancer (TNBC).^[^ ^95^ ^]^ These studies underscore the pivotal role of tissue‐resident T cells in effective ICI responses.

In addition to tissue‐resident phenotypes, T cell exhaustion features are also closely linked to ICI responses. Zhang et al. identified *CXCL13* ^+^ T cells, expressing exhaustion markers and comprising both CD4^+^ and CD8^+^ subsets, are enriched in ICI‐responsive TNBC patients.^[^ ^96^ ^]^ Similarly, Bassez et al. demonstrated that PD‐1^+^ CD8^+^ T cells, characterized by exhaustion markers, cytotoxic markers, and immune cell homing markers such as *CXCL13*, underwent clonal expansion following anti‐PD‐1 treatment in breast cancer.^[^ ^97^ ^]^ In colorectal cancer (CRC), Chen et al. observed that Tex and tumor‐reactive‐like (Ttr‐like) CD8^+^ T cells are strongly associated with responsiveness to anti‐PD‐1 therapy.^[^ ^98^ ^]^ These findings highlight the relevance of exhaustion‐associated features in ICI responses. Notably, tissue‐resident and exhaustion phenotypes may overlap,^[^ ^99^ ^]^ and further studies are needed to elucidate their interplay and lineage relationships within the TME.

One key question in ICI therapies is whether the T cell response relies on reinvigorating pre‐existing TILs or recruiting T cells from peripheral tissues. TCRs, with their diverse repertoires, can serve as tags or barcodes to track T cell lineages and dynamics during ICIs treatment. Using scRNA‐seq and scTCR‐seq on site‐matched tumors from patients with basal or squamous cell carcinoma (BCC or SCC) before and after anti‐PD‐1 therapy, Yost et al. demonstrated that PD‐1 blockade primarily drives clonal replacement, particularly in CD8^+^ Tex cells, rather than the reinvigoration of pre‐existing TILs.^[^ ^100^ ^]^ In contrast, Zhang et al. observed that in TNBC, pre‐existing TCR clones clonally expand in tumors responsive to ICIs and identified *CXCL13* ^+^ T cells as potential tumor‐reactive T cells responsive to anti‐PD‐L1 blockade.^[^ ^96^ ^]^ In lung cancer, Liu et al. observed an increase in progenitor Tex (Tpex) cells and expanded new and pre‐existing clonotypes in tumors responding to anti‐PD‐1 therapy, concluding that Tpex cells accumulate through both local expansion and replenishment by peripheral T cells, a phenomenon termed “clonal revival.”^[^ ^101^ ^]^ Au et al. demonstrated that nivolumab induces both the maintenance and replacement of pre‐expanded T cell clones, with only the maintenance of these clones correlating with therapeutic response.^[^ ^102^ ^]^ These studies suggest that both pre‐existing and newly infiltrated clones, depending on the specific cancer type or TME, contribute to the effectiveness of ICI responses.

### 3.3 Importance of Progenitor and Stem‐Like TILs in Response to ICIs

Advances in single‐cell technologies have deepened our understanding of T cell states within tumors and their variability across clinical contexts. Miller et al. identified distinct subsets of CD8^+^ Tex cells with varying roles in tumor control and ICI responses, reporting that only “progenitor exhausted” TILs, which retain polyfunctionality and persist long‐term, respond to anti‐PD‐1 therapy and correlate with prolonged responses in melanoma.^[^ ^103^ ^]^ Likewise, Liu et al. identified a subset of CD8^+^ Tex cells expressing *SPRY1*, which exhibits a Tpex phenotype and is associated with complete responses to neoadjuvant PD‐1 blockade in esophageal squamous cell carcinoma (ESCC).^[^ ^104^ ^]^ Notably, Siddiqui et al. reported that intratumoral Tcf1^+^ PD‐1^+^ CD8^+^ T cells with stem‐like properties are crucial for tumor control in response to ICIs, both in mouse models and human tumors.^[^ ^105^ ^]^ Kurtulus et al. demonstrated that TILs with effector‐ and memory‐precursor‐like properties expand in response to ICI therapies and exhibite both proliferative and effector capacities, dependent on Tcf7/Tcf1 expression.^[^ ^106^ ^]^ Consistently, Sade‐Feldman et al. identified two distinct CD8^+^ T cell states in melanoma associated with ICI responses, where *TCF7* expression predicts positive clinical outcomes, while dysfunctional signatures correlate with ICI failure.^[^ ^107^ ^]^ Bi et al. found that a subpopulation of CD8^+^ stem‐like T cells is activated and differentiates into a terminally exhausted phenotype in responders to ICI treatment in metastatic renal cell carcinoma (RCC).^[^ ^108^ ^]^ These findings underscore the critical role of progenitor and stem‐like TILs in mediating effective ICI responses, with the transcription factor TCF7/TCF1 serving as a key marker of these functionally important subsets.

Concordantly, Eberhardt et al. profiled CD8^+^ T cells in human papillomavirus (HPV)‐positive head and neck cancer, and identified HPV‐specific TCF‐1^+^ PD‐1^+^ stem‐like TILs that proliferate and differentiate into effector‐like cells upon HPV peptide stimulation and PD‐1 blockade.^[^ ^109^ ^]^ Similar results have been observed in chronic infection models. Im et al. identified a subset of virus‐specific Tcf1^+^ CD8^+^ T cells with features of CD4^+^ T follicular helper (Tfh) cells, CD8^+^ memory precursors, and hematopoietic stem cell progenitors, which drive the proliferative burst after PD‐1 therapy in LCMV‐infected mice.^[^ ^110^ ^]^ Utzschneider et al. identified virus‐specific Tcf1^+^ CD8^+^ T cells with memory‐like phenotype that are essential for T cell expansion in response to ICIs during chronic infection.^[^ ^111^ ^]^ These findings underscore the critical role of Tcf1⁺ CD8⁺ T cells, often referred to as ‘progenitor exhausted’ or stem‐like cells, in sustaining immune responses and mediating effective ICI therapy (Figure 1).

### 3.4 Critical Role of CD4^+^ T Cells in Anti‐Tumor Immunity and ICI Responses

Although CD8^+^ T cells have traditionally been the central focus, CD4^+^ T cells are increasingly recognized for their essential and multifaceted roles in anti‐tumor immunity and ICI responses.^[^ ^112^, ^113^, ^114^ ^]^ Linnemann et al. showed that, in addition to the well‐established recognition of neoantigens by CD8^+^ T cells, neoantigen‐specific CD4^+^ T cells are also frequently present in human tumors.^[^ ^115^ ^]^ Consistently, Oh et al. found that intratumoral CD4^+^ T cells, specifically clonally expanded cytotoxic subsets, mediate anti‐tumor immunity in bladder cancer.^[^ ^116^ ^]^ Quezada et al. reported that naive tumor‐reactive CD4^+^ T cells can drive substantial T cell expansion and tumor regression through class II‐restricted recognition of tumor antigens.^[^ ^117^ ^]^ Alspach et al. also emphasized that MHC class II‐restricted antigen expression on tumor cells is crucial for successful tumor rejection, both spontaneously and in response to ICI treatment.^[^ ^118^ ^]^ Additionally, Spitzer et al. demonstrated that CD4^+^ T cells alone are sufficient to initiate immune responses, highlighting their crucial role in immune activation.^[^ ^119^ ^]^ These findings collectively underscore the pivotal role of CD4^+^ T cell activation in mediating effective anti‐tumor responses.

**Figure 2:**
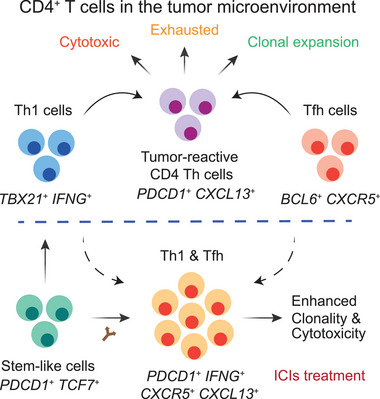
Characteristics of key CD4^+^ T cell subsets associated with ICI responses. Tumor‐reactive CD4^+^ Th cells, characterized by *PDCD1* and *CXCL13*, exhibit both exhausted and cytotoxic features while maintaining a high proliferative capacity. These cells show features of both Th1 and Tfh. Following ICI treatment, responsive CD4^+^ Th cells expressing *PDCD1*, *IFNG*, *CXCR5*, and *CXCL13* exhibit enhanced clonality and cytotoxicity, potentially originating from stem‐like *TCF7* ^+^ precursor cells.

CD4^+^ T cells consist of heterogeneous subsets, with the presence of Th1 cells generally correlating with improved clinical outcomes.^[^ ^120^, ^121^, ^122^ ^]^ Tran et al. showed that mutation‐specific CD4^+^ Th1 cells can reduce target lesions and prolong disease stabilization in a patient with metastatic cholangiocarcinoma.^[^ ^123^ ^]^ In line with this, Xie et al. showed that tumor‐specific naïve CD4^+^ T cells differentiate into Th1 cells in vivo, effectively eradicating established melanoma.^[^ ^124^ ^]^ Similarly, Jiao et al. found that ICI treatment significantly enhances the infiltration of intra‐tumoral Th1 cells, leading to improved survival outcomes, while the absence of Th1 cells contributes to resistance to ICIs in a subcutaneous castration‐resistant prostate cancer (CRPC) model.^[^ ^125^ ^]^ Given the complexity of T cell states in the TME, refined definitions, especially based on signature expression, have been established to better characterize these subsets. Cohen et al. identified CD4^+^ PD‐1^+^ CXCL13^+^ T cells as a critical hub for interactions with antigen‐presenting cells in the TME of NSCLC.^[^ ^126^ ^]^ Cardenas et al. characterized a population of CD4^+^ PD‐1^+^ TCF1^+^ T cells with stem‐like properties, capable of self‐renewal and differentiation into Th1 cells, which enhance tumor control and improve responsiveness to immunotherapy.^[^ ^127^ ^]^ Similarly, Bassez et al. showed that anti‐PD1 treatment in breast cancer triggers the clonal expansion of CD4^+^ T cell subsets expressing Th1 markers like *IFNG*, alongside Tfh cell markers such as *BCL6* and *CXCR5* ^97^. Zhang et al. observed an expansion of CD4^+^ CXCL13^+^ T cells, which resemble both Th1 and Tfh cells, following ICI‐based therapies in TNBC.^[^ ^96^ ^]^ Additionally, Yost et al. found that ICI treatment in BCC and SCC increases the frequency of CXCR5^+^ Tfh cells, along with enhanced clonality within this population.^[^ ^100^ ^]^ These findings suggest that specific CD4^+^ T cells, which exhibit features of both Th1 and Tfh cells, play a pivotal role in anti‐tumor immunity and contribute to ICI responses (**Figure** **2**). Further studies indicate that these cells also display characteristics of Tex and Trm,^[^ ^128^, ^129^ ^]^ and the precise relationships and differentiation pathways among these subsets remain to be fully elucidated.

## 4 Immune Cell Characteristics in Peripheral Blood Associated with ICI Responses

**Figure 3:**
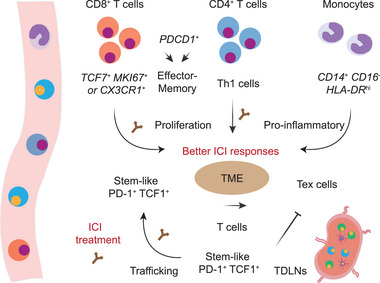
Immune cell characteristics and dynamics in peripheral blood and tumor‐draining lymph nodes associated with ICI responses. In peripheral blood, CD8^+^ T cells expressing PD‐1, TCF1, MKI67, or CX3CR1 are associated with favorable ICI responses. Similarly, CD4^+^ Th1 cells correlate with improved outcomes. Both CD8^+^ and CD4^+^ T cells exhibit effector and memory phenotypes and undergo proliferation following ICI therapies. Pro‐inflammatory monocytes characterized by CD14^+^ CD16^−^ HLA‐DR^hi^ are associated with enhanced ICI responses. In TDLNs, stem‐like PD‐1^+^ TCF1^+^ T cells do not differentiate into Tex cells but instead expand and migrate into tumors following ICI therapies, thereby contributing to sustained anti‐tumor immunity.

Although studies have largely focused on local immune responses within the TME, effective anti‐tumor immunity relies on coordinated responses across various tissues and cell types. Spitzer et al. emphasized the significance of systemic immune responses in tumor eradication, demonstrating that peripheral immune cells are pivotal in sustaining proliferation during tumor rejection following successful ICI therapies.^[^ ^119^ ^]^ This underscores the critical role of peripheral immune cells in driving effective anti‐tumor immunity and mediating responses to ICIs (**Figure** **3**).

Peripheral blood plays a crucial role in monitoring immune cell activity, contributing to the activation, proliferation, and trafficking of key immune cells. Changes in the composition and activation status of immune cells in the blood reflect, at least in part, the immune cell dynamics within the TME, providing valuable insights into treatment responses and correlating with clinical outcomes. Gros et al. identified neoantigen‐specific lymphocytes in the peripheral blood of melanoma patients and demonstrated that circulating CD8^+^ PD‐1^+^ lymphocytes closely resemble the tumor‐resident anti‐tumor lymphocyte population.^[^ ^130^ ^]^ Similarly, Siddiqui et al. identified TCF1^+^ PD‐1^+^ cells in both the blood and TILs of melanoma patients, which display characteristics of both exhausted and central memory T (Tcm) cells and mediate the proliferative response to immunotherapy.^[^ ^105^ ^]^ Yamauchi et al. showed that an early increase in circulating CX3CR1^+^ CD8^+^ T cells following anti‐PD‐1 therapy correlates with better treatment response and survival in NSCLC.^[^ ^131^ ^]^ Likewise, Kamphorst et al. observed an increase in Ki‐67^+^ PD‐1^+^ CD8^+^ T cells in the peripheral blood of patients with advanced NSCLC undergoing PD‐1 blockade therapy, with most responses occurring after the first or second treatment cycle.^[^ ^132^ ^]^ Fairfax et al. observed that in responders with metastatic melanoma, peripheral CD8^+^ T cells exhibit a higher proportion of effector T (Teff) or memory T (Tmem) cells and larger TCR clones, both of which are associated with sustained treatment efficacy.^[^ ^133^ ^]^ These findings suggest that T cell populations and TCR clonality in peripheral blood are closely linked to intratumoral immune infiltration and responsiveness to ICIs. Similarly, Chen et al. observed that peripheral T cells in responders to PD‐1 blockade in CRC exhibit higher diversity of TCR clones.^[^ ^98^ ^]^ Wu et al. demonstrated that the expansion of peripheral T cells correlates with tumor infiltration and predicts clinical response and is associated with clonotypes originally detected in both tumor and adjacent normal tissues.^[^ ^134^ ^]^ These studies suggest that peripheral T cells are indicative of ICI responses.

However, the relationship between peripheral T cells and TILs remains complex. Krieg et al. characterized immune cell subsets in the peripheral blood of melanoma patients, observing a reduction in peripheral T cells alongside an increase in tumor‐infiltrating CD8^+^ T cells in responders to ICIs.^[^ ^135^ ^]^ Oliveira et al. noted that the persistence of TCR clonotypes in peripheral blood negatively correlates with intratumoral exhaustion and exhibits higher levels in patients with poor responses to ICIs, indicative of chronic stimulation by residual tumor antigens.^[^ ^87^ ^]^ These findings hint at the potential opposing dynamics between peripheral T cells and TILs. Collectively, these studies suggest that specific circulating lymphocyte subsets can serve as indicators of TIL populations, reflecting the immune response within the TME.

Along with CD8^+^ T cells, CD4^+^ T cells, and monocytes have also emerged as important peripheral blood components for predicting responses to ICI therapies. Kitano et al. demonstrated that, after ICI treatment, tumor antigen‐specific CD4^+^ T cell responses are either induced or enhanced in the peripheral blood of patients with advanced melanoma, with a dominant Th1 immune profile observed.^[^ ^136^ ^]^ Spitzer et al. similarly identified a subset of peripheral CD4^+^ T cells with an activated, effector‐memory Th1 phenotype, which are crucial in protecting against new tumors, and exhibit expansion in responders to immunotherapy.^[^ ^119^ ^]^ Krieg et al. found that the frequency of CD14^+^ CD16^−^ HLA‐DR^hi^ monocytes in peripheral blood is a strong predictor of survival in response to anti‐PD‐1 therapy in melanoma patients.^[^ ^135^ ^]^ Similarly, Zhang et al. observed that the transcriptional characteristics of monocytes in the peripheral blood of TNBC patients partially mirror those in the TME, noting that monocytes from responders display a pro‐inflammatory profile, whereas those from non‐responders show an anti‐inflammatory profile.^[^ ^96^ ^]^

## 5 Immune Cell Dynamics in Tumor‐Draining Lymph Nodes and Their Interaction with the TME

According to the cancer‐immunity cycle, tumor‐draining lymph nodes (TDLNs) are pivotal in orchestrating immune surveillance and anti‐tumor response.^[^ ^1^, ^137^, ^138^ ^]^ Previous studies have underscored the strong interplay between the immune response within the TME and the broader systemic immune landscape, with TDLNs serving as crucial sites for the activation of anti‐tumor T cells. In preclinical tumor models, Huang et al. identified a subset of tumor‐specific CD8^+^ T cells in the TDLNs that retain memory‐like characteristics instead of undergoing functional exhaustion, which exhibit enhanced anti‐tumor efficacy following adoptive transfer and are responsive to ICIs.^[^ ^139^ ^]^ Similarly, Schenkel et al. found that, unlike intratumoral TCF‐1^+^ CD8^+^ T cells that become dysfunctional and decline in number as tumors progress, the frequency of TCF‐1^+^ CD8^+^ T cells in TDLNs remains stable. This stability is supported by conventional type I dendritic cells (cDC1), which help maintain a reservoir of proliferative tumor‐antigen‐specific TCF‐1^+^ CD8^+^ T cells.^[^ ^140^ ^]^

Regarding the connection between immune cells in TDLNs and the TME, as well as their implications in ICI therapies, Dammeijer et al. showed in mouse models that TDLNs are enriched with tumor‐specific PD‐1^+^ T cells, and PD‐L1 blockade targeting TDLNs enhances anti‐tumor T cell immunity by mobilizing Tpex to the tumor site, leading to improved tumor control.^[^ ^141^ ^]^ Prokhnevska et al. examined human TDLNs and found that activated CD8^+^ T cells in these nodes share functional, transcriptional, and epigenetic features with TCF1^+^ stem‐like cells in the tumor, suggesting that these TDLN‐derived cells serve as precursors to tumor‐resident stem‐like CD8^+^ T cells.^[^ ^142^ ^]^ Similarly, Connolly et al. identified a reservoir of stem‐like CD8^+^ T cells in TDLNs, with functional and gene expression profiles resemble “stem‐like” TCF1^+^ cells in the TME. These TDLN T cells are developmental precursors to, and clonally related to, their more differentiated intratumoral counterparts, suggesting continuous replenishment through migration.^[^ ^143^ ^]^ Notably, Rahim et al. identified CD8^+^ Tpex in uninvolved lymph nodes of head and neck squamous cell carcinoma patients, which are clonally related to tumor‐resident terminal Tex.^[^ ^144^ ^]^ Following ICI treatment, the Tpex population decreases and clusters near DCs, suggesting activation. In line with these findings, Li et al. used in vivo labeling to demonstrate the continuous trafficking of TCF‐1^+^ T cells between the tumor and TDLNs.^[^ ^145^ ^]^ These results suggest that specific T cell subsets in TDLNs, particularly those with stem‐like properties marked by TCF1/TCF7, can further differentiate and infiltrate into tumors, thereby continuously replenishing the T cell pool in the TME (Figure 3).

## 6 Crucial Role of B Cells in the TME and Their Impact on ICI Therapies

Although T cells are central to anti‐tumor immune responses and serve as the primary targets of ICIs, other immune cells also play critical roles in regulating T cell activity.^[^ ^137^ ^]^ Increasing evidence underscores the contribution of B cells to the efficacy of ICIs. Petitprez et al. demonstrated that the presence of B cells is associated with improved survival and enhanced responses to ICIs in sarcoma.^[^ ^146^ ^]^ Likewise, Helmink et al. found that B cells contribute to favorable immunotherapy outcomes, with clonal expansion and distinct functional states observed in melanoma patients responding to ICIs.^[^ ^24^ ^]^ Notably, B cells are often found in conjunction with T cells. Cabrita et al. reported that the coexistence of tumor associated CD8^+^ T cells and CD20^+^ B cells in metastatic melanoma correlates with improved survival and predicts positive responses to ICIs.^[^ ^147^ ^]^ Zhang et al. observed that follicular or germinal center (GC) B cells are strongly associated with *CXCL13* ^+^ T cells, which concertedly expand after ICI treatment and predict favorable outcomes in TNBC.^[^ ^96^ ^]^ Additionally, Hollern et al. found that ICI treatment activates Tfh and B cells, with B cells contributing to anti‐tumor immune responses by secreting antibodies and activating T cells in mouse models of TNBC.^[^ ^148^ ^]^ Li et al. showed that PD‐1 blockade in mismatch repair‐deficient CRC reduces CD4^+^ regulatory T (Tregs) cells and increases CD20^+^ B cells, which are linked to a higher abundance of CD8^+^ effector‐memory T (Tem) cells in patients who achieved a pathological complete response (pCR) to anti‐PD‐1 therapy.^[^ ^149^ ^]^ Collectively, these studies indicate that B cells not only function as predictive biomarkers for favorable responses to ICIs but also play a direct mechanistic role in enhancing immunotherapy efficacy.

**Figure 4:**
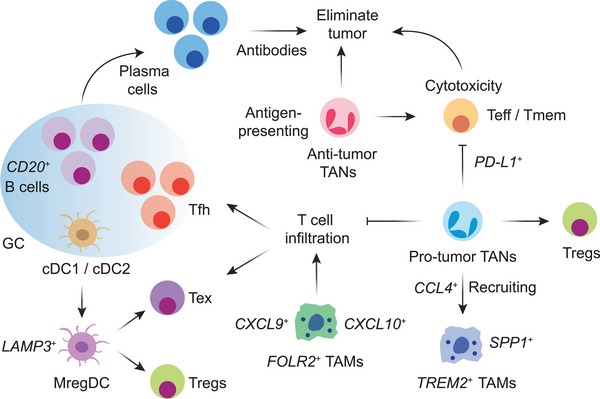
Dynamic interactions among various immune cell populations. B cells exert their functions through antibody production and T cell activation, primarily by interacting with Tfh cells. DCs could transit into mregDCs, which regulate Tregs and Tex cells. *CXCL9* ^+^/*CXCL10* ^+^ and *FOLR2* ^+^ macrophages promote T cell infiltration and are associated with favorable responses to ICIs, while *TREM2* ^+^ macrophages exhibit immunosuppressive properties, correlating with poor prognosis and reduced ICI efficacy. TANs can exhibit either pro‐ or anti‐tumor effects. Pro‐tumor TANs suppress Teff and Tmem cells, recruit Tregs, support *TREM2* ^+^ macrophages, and inhibit T cell infiltration into the TME. Anti‐tumor TANs directly eliminate cancer cells, present antigens, and enhance T cell responses.

Although the precise mechanisms underlying B cell‐mediated modulation of ICI efficacy remain to be fully elucidated, emerging evidence implicates their involvement in antibody production, antigen presentation, and T cell response regulation^[^ ^150^, ^151^, ^152^ ^]^ (**Figure** **4**). Additionally, the role of B cells in chemotherapy responses has also been explored, identifying an *ICOSL* ^+^ B cell subset following treatment that enhances anti‐tumor immunity by increasing the effector‐to‐regulatory T cell ratio.^[^ ^153^ ^]^ Single‐cell sequencing has unveiled substantial heterogeneity within B cell populations,^[^ ^154^, ^155^ ^]^ highlighting the need for further research to fully elucidate the distinct functions of B cell subsets and their influence on anti‐tumor immune responses and therapeutic outcomes.

## 7 Involvement of Myeloid Cells in Anti‐Tumor Immunity and Their Impact on ICIs

Myeloid cells have emerged as pivotal modulators of T cell responses within the TME.^[^ ^156^ ^]^ Braun et al. found that Tex and M2‐like tumor‐associated macrophages (TAMs) are enriched in tumors, where their interactions form a detrimental immune dysfunction circuit that impairs the anti‐tumor immune response and is associated with poorer prognosis in advanced ccRCC.^[^ ^157^ ^]^ Zhang et al. demonstrated that *TREM2* ^+^ macrophages are immunosuppressive and associated with poor prognosis and reduced responses to anti‐PD‐1 therapy in NSCLC.^[^ ^158^ ^]^ Moreover, *TREM2* ^+^ macrophages were shown to inhibit CD8^+^ T‐cell infiltration in hepatocellular carcinoma (HCC).^[^ ^159^ ^]^ In contrast, pro‐inflammatory macrophages, characterized by *CXCL9* and *CXCL10*, were found to be closely associated with *CXCL13* ^+^ T cells and IFN‐γ responses in TNBC, suggesting active anti‐tumor immunity within the TME.^[^ ^96^ ^]^ Additionally, Ramos et al. identified tissue resident *FOLR2* ^+^ macrophages in human breast cancer, located in the perivascular regions of the tumor stroma, where they interact with and promote CD8^+^ T cell infiltration.^[^ ^160^ ^]^ Together, these findings underscore the multifaceted roles of macrophages in shaping the immune landscape (Figure 4), which critically influence the effectiveness of immunotherapy.

Following ICI therapy, Gao et al. observed upregulation of PD‐L1 and VISTA on macrophage subsets in prostate tumors, and identified VISTA as a potential inhibitory immune checkpoint, highlighting the pivotal role of macrophages in modulating ICI responses.^[^ ^161^ ^]^ Gubin et al. observed that intratumoral myeloid cells exhibit a spectrum of activation states, ranging from anti‐inflammatory in progressively growing tumors to pro‐inflammatory in tumors successfully rejected in ICI‐treated mice.^[^ ^162^ ^]^ Similarly, Bi et al. found that macrophages shift to pro‐inflammatory states in response to an interferon‐rich microenvironment after ICI exposure, while simultaneously upregulating immunosuppressive markers in patients with advanced RCC.^[^ ^108^ ^]^ Krishna et al. identified interactions between TAMs and CD8^+^ tissue‐resident T cells, which may contribute to pro‐inflammatory responses in patients with complete responses to ICIs.^[^ ^93^ ^]^ These observations suggest a phenotypic shift in macrophages following effective ICI treatment, likely driven by an active TME shaped by T cell responses. This shift may, in turn, enhance anti‐tumor immunity and contribute to therapeutic success.

In addition to macrophages, DCs function in antigen presentation and are essential for T cell activation. Broz et al. found that *CD103* ^+^ DCs, though sparse, are highly effective in stimulating cytotoxic T lymphocytes (CTLs).^[^ ^163^ ^]^ Ferris et al. showed that tumor rejection relies on cDC1, with CD8^+^ T cell priming dependent on MHC class I expression by cDC1. They also revealed that cDC1 are essential for the early priming of CD4^+^ T cells against tumor‐derived antigens.^[^ ^164^ ^]^ In a related study, Pasqual et al. employed intercellular enzymatic labeling to monitor in vivo interactions between DCs and T cells, revealing that DCs are essential for priming CD4^+^ T cells during immune responses.^[^ ^165^ ^]^ Consistently, Cohen et al. demonstrated that interactions between CD4^+^ Th cells and DCs are crucial for shaping the effective anti‐tumor immunity and mediating the ICI responses.^[^ ^126^ ^]^ However, within the TME, the phenotype and function of DCs can be altered. Oh et al. found that DCs in the TME are a major source of PD‐L1, despite being outnumbered by PD‐L1^+^ macrophages, and that selectively deleting PD‐L1 from DCs, but not macrophages, boosts anti‐tumor CD8^+^ T cell responses and inhibits tumor growth.^[^ ^166^ ^]^ Concordantly, Dammeijer et al. showed that tumor‐specific PD‐1^+^ T cells interact closely with PD‐L1^+^ cDCs in TDLNs.^[^ ^141^ ^]^ Notably, Maier et al. identified a subset of DCs, termed mregDCs (mature and regulatory dendritic cells), which co‐express immunoregulatory and maturation‐associated genes and suppress T cell responses in NSCLC.^[^ ^167^ ^]^ Zhang et al. identified a similar subset of DCs, named as *LAMP3* ^+^ DCs, in human HCC, which could migrate from primary tumors to hepatic lymph nodes and regulate various lymphocyte subsets, including Tex and Tregs.^[^ ^31^ ^]^

Under ICI treatment, Zhang et al. reported a notable increase in DC representation in the TME of TNBC patients who responded to anti‐PD‐L1 therapies.^[^ ^96^ ^]^ Salmon et al. demonstrated that inadequate activation of *CD103* ^+^ DCs limits ICI effectiveness, underscoring the critical role of these DCs in mediating anti‐tumor responses following PD‐L1 blockade in melanoma mouse models.^[^ ^168^ ^]^ Garris et al. found that tumor‐infiltrating DCs producing interleukin (IL)‐12 are crucial for robust anti‐tumor responses, with T cell‐DC crosstalk, mediated by IFN‐γ and IL‐12, being vital for the success of anti‐PD‐1 therapy.^[^ ^169^ ^]^ In addition to cDC1, cDC2 is also involved in ICI therapies. Binnewies et al. observed that, in melanoma patients with low Treg abundance, intratumoral cDC2 density correlates with abundant CD4^+^ conventional T (Tconv) cells and improved responsiveness to anti‐PD‐1 therapy.^[^ ^170^ ^]^

## 8 Growing Focus on Neutrophils and Their Roles in Orchestrating Anti‐Tumor Immunity

Neutrophils are now recognized as pivotal regulators of anti‐tumor immunity, though their functional contributions have historically been underappreciated, likely due to technical challenges in capturing them, as tumor‐associated neutrophils (TANs) are short‐lived and sensitive to tissue processing.^[^ ^171^, ^172^ ^]^ Despite this, emerging evidence highlights neutrophils as crucial mediators of the immunosuppressive environment and as drivers of poor prognosis and ICI resistance (Figure 4). Wang et al. characterized the heterogeneity of TANs and identified a pro‐tumor subpopulation (TAN‐1) that is linked to poor prognosis in pancreatic ductal adenocarcinoma (PDAC).^[^ ^173^ ^]^ Similarly, Xue et al. investigated neutrophil heterogeneity in HCC, finding that myeloid‐enriched TANs correlate with poor prognosis, with *CCL4* ^+^ TANs recruiting macrophages and *PD‐L1* ^+^ TANs inhibiting T cell cytotoxicity.^[^ ^174^ ^]^ Salcher et al. analyzed tissue‐resident neutrophils (TRNs) in the NSCLC microenvironment, uncovering that the TRN signature is linked to ICI resistance.^[^ ^175^ ^]^ Consistently, Steele et al. demonstrated that inhibiting CXCR2 signaling, which is predominantly upregulated in neutrophils and myeloid‐derived suppressor cells (MDSCs), profoundly suppresses metastasis and enhances the efficacy of immunotherapy in PDAC.^[^ ^176^ ^]^ Notably, tumors with low TILs often harbor higher proportions of TANs and show resistance to ICIs, while disrupting key signaling pathways that reduce TAN infiltration, such as CXCL1 or CXCL5/CXCR2 signaling, increases CD8^+^ PD‐1^+^ T cell levels, and improves tumor sensitivity to ICI therapy.^[^ ^177^, ^178^ ^]^

The immune suppression by neutrophils involves several mechanisms.^[^ ^179^, ^180^ ^]^ Taifour et al. found that Chi3l1 expression in epithelial cancers facilitates neutrophil recruitment and the formation of neutrophil extracellular traps (NETs), which promote T cell exclusion to the stroma and inhibit T cell infiltration.^[^ ^181^ ^]^ Teijeira et al. showed that neutrophils and granulocytic MDSCs from cancer patients extrude their NETs, which encase tumor cells and shield them from cytotoxicity by CD8^+^ T cells and natural killer (NK) cells, thereby obstructing immune cell‐target cell interactions.^[^ ^182^ ^]^ Wang et al. demonstrated that NETs bridge innate and adaptive immunity by promoting Treg differentiation through metabolic reprogramming of naïve CD4^+^ T cells.^[^ ^183^ ^]^ Kim et al. found that pathologically activated neutrophils, termed polymorphonuclear (PMN) MDSCs, are susceptible to ferroptosis, which boosts their immunosuppressive activity by converting non‐suppressive PMNs into PMN‐MDSCs and increasing the release of molecules that inhibit T cell activity.^[^ ^184^ ^]^ Concordantly, Veglia et al. showed that inhibiting fatty acid transport protein 2 (FATP2) abrogates the activity of PMN‐MDSCs and substantially delays tumor progression.^[^ ^179^, ^180^ ^]^ Moreover, TANs can inhibit T cell proliferation and induce T cell apoptosis through multiple molecules, such as reactive oxygen species (ROS), nitric oxide (NO), Fas ligand, and TRAIL.^[^ ^185^, ^186^, ^187^ ^]^ These findings suggest that TANs suppress anti‐tumor immune responses through multiple mechanisms, including the formation of NETs and metabolic reprogramming.

Contrary to their conventional pro‐tumor characteristics, recent studies have revealed an immunostimulatory subset of TANs with potent anti‐tumor capabilities. Wu et al. revealed an antigen‐presenting neutrophil population that correlates with improved survival and shows dual capacity to prime both tumor‐specific (via neoantigen presentation) and broad‐spectrum (through antigen‐independent mechanisms) T cell responses.^[^ ^188^ ^]^ Gungabeesoon et al. showed that CD40 agonist treatment rapidly expands a specialized neutrophil subset exhibiting an interferon‐stimulated gene (ISG) signature linked to better clinical outcomes.^[^ ^189^ ^]^ Notably, Hirschhorn et al. revealed coordinated anti‐tumor activity between T cells and neutrophils during immunotherapy, where tumor‐specific CD4^+^ T cells initiate responses against melanoma antigens while neutrophils eliminate antigen‐loss variants.^[^ ^190^ ^]^ Consistently, Cui et al. demonstrated that tumor‐infiltrating neutrophils exert direct anti‐tumor effects through secretion of catalytically active neutrophil elastase, which selectively induces cancer cell death, inhibits primary tumor progression, and elicits systemic anti‐tumor immunity by activating CD8^+^ T cell‐dependent abscopal effects against metastatic lesions.^[^ ^191^ ^]^ Similarly, Faraoni et al. found that neutrophils mediate abscopal immunomodulation after radiofrequency ablation in pancreatic cancer, thereby promoting anti‐tumor immunity.^[^ ^192^ ^]^ These findings suggest that specific subsets of neutrophils can elicit anti‐tumor immunity and mediate effective responses in ICI therapies (Figure 4). Single‐cell analyses revealed that neutrophils display remarkable functional plasticity, demonstrating both pro‐ and anti‐tumor activities that are highly context‐dependent.^[^ ^174^, ^188^, ^193^, ^194^, ^195^ ^]^ Current findings underscore the need for more precise neutrophil subpopulation characterization and deeper investigation of their dual roles in tumor progression, immune surveillance, and response to ICIs.

## 9 Coordinated Dynamics of Key Anti‐Tumor Immune Cells in Response to ICI Therapies

Effective anti‐tumor immunity requires tightly coordinated interactions among diverse immune cell populations within the tumor immune microenvironment (TIME). Bindea et al. systematically mapped immune cell networks in CRC, demonstrating synchronized immunome‐wide gene expression patterns and revealing positive correlations between markers of distinct immune subsets.^[^ ^196^ ^]^ Specifically, they identified a network of intratumoral T, Tfh, and B cells, highlighting the functional co‐modulation between these populations. Similarly, Pelka et al. performed single‐cell analyses of MMR‐deficient and ‐proficient CRC specimens, discovering spatially organized immune hubs and defining conserved multicellular functional modules.^[^ ^21^ ^]^ These findings emphasize the coordinated function of immune cells within the TME, with multiple interconnected immune subsets playing crucial roles in anti‐tumor responses.

Intervention‐based studies have also highlighted the coordinated modulation of immune cell subsets in response to ICI therapies. Zhang et al. observed a concerted increase in lymphoid tissue inducer (LTi) cells, follicular B cells, *CXCL13* ^+^ T cells, and cDC1 in TNBC patients who responded to ICI combination therapy.^[^ ^96^ ^]^ Similarly, Chen et al. identified locally coordinated cellular programs in human CRC following PD‐1 blockade, noting coupled changes between Tex cells and multiple tumor‐enriched populations, including *4‐1BB* ^+^ Treg, *LAMP3* ^+^ DC, and IgG^+^ plasma cells.^[^ ^98^ ^]^ Li et al. observed that in MMR‐deficient/microsatellite instability‐high (MSI‐H) CRCs, tumors achieving pCR after neoadjuvant PD‐1 blockade exhibit a coordinated reduction in CD8⁺ Trm‐mitotic cells, CD4⁺ Tregs, *IL1B*⁺ monocytes, and *CCL2*⁺ fibroblasts, alongside an increase in CD8⁺ Tem, CD4⁺ Th, *CD20*⁺ B cells, and *HLA‐DRA*⁺ endothelial cells.^[^ ^149^ ^]^ Davar et al. investigated combined vidutolimod (TLR9 agonist) and nivolumab (PD‐1 inhibitor) therapy in high‐risk resectable melanoma, demonstrating that major pathological response (MPR) is associated with tumor necrosis, melanophagocytosis, and an increase in CD8⁺ TILs and plasmacytoid dendritic cells (pDCs) in the TME.^[^ ^197^ ^]^ These coordinated immune cell alterations reflect pro‐inflammatory reprogramming of the TME following effective ICIs.

## 10 Involvement of Stromal Components in Anti‐Tumor Immunity and ICI Responses

In addition to immune cells, other stromal components can profoundly influence immune‐tumor interactions and, consequently, tumor responses to ICIs.^[^ ^13^, ^198^, ^199^ ^]^ Among these, cancer‐associated fibroblasts (CAFs) are the most abundant cell type in solid tumors and are key drivers of tumor growth and metastasis.^[^ ^20^, ^200^, ^201^, ^202^ ^]^ Mariathasan et al. identified TGFβ signaling in fibroblasts as a critical factor contributing to the lack of response to ICIs in metastatic urothelial cancer, particularly in tumors where CD8^+^ T cells are excluded and localized to the peritumoral stroma.^[^ ^203^ ^]^ Moreover, co‐administration of TGFβ‐blocking and anti‐PD‐L1 antibodies reduces TGFβ signaling, enhances T cell infiltration, and triggers tumor regression.^[^ ^204^ ^]^ These findings position CAFs as promising therapeutic targets within the TME.

**Figure 5:**
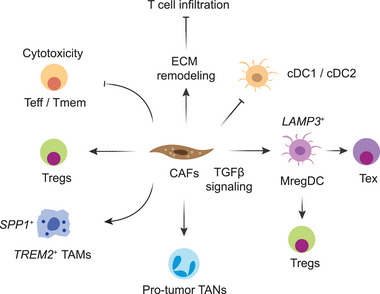
Regulatory roles of CAFs in the TME. CAFs play multifaceted immunoregulatory roles in the TME. They remodel the ECM, creating physical barriers that restrict T cell infiltration. CAFs also recruit immunosuppressive populations, including Tregs and *TREM2*⁺ macrophages, and directly inhibit T cell activation. Moreover, they impair DC function by promoting dysfunctional or tolerogenic DC subsets, which further support Treg expansion and T cell exhaustion.

CAFs interact with both cancer and immune cells, driving extracellular matrix (ECM) remodeling and modulating the immune response^[^ ^20^, ^205^, ^206^, ^207^, ^208^ ^]^ (**Figure** **5**). The mechanisms by which CAFs regulate immune cells and impact ICI responses are complex, involving multiple layers of interaction with various components of the TME. As one of major components of the tumor stroma, CAFs play a critical role in ECM remodeling by secreting matrix proteins like type I collagen, fibronectin, and MMP‐1, along with releasing cytokines, resulting in the formation of a permeability barrier that limits T cell infiltration and contributes to CD8^+^ T cell depletion.^[^ ^201^, ^205^, ^209^ ^]^ CAFs have also been shown to promote the recruitment and migration of Tregs, and induce CD8^+^ T cells to express Foxp3, thereby increasing the accumulation of immunosuppressive Tregs at tumor sites.^[^ ^202^, ^210^, ^211^, ^212^ ^]^ Additionally, CAFs promote the recruitment of monocytes and drive their differentiation into M2 macrophages, which suppress Teff cell responses and contribute to immune suppression within the TME.^[^ ^213^, ^214^, ^215^ ^]^ CAFs also recruit neutrophils into tumors by secreting stromal‐derived factor‐1 alpha (SDF‐1α) and CXCR2, which guide TAN migration.^[^ ^194^, ^216^ ^]^ Moreover, CAFs release cytokines like cardiotrophin‐like cytokine 1 (CLCF1), which modulate TAN polarization and promote tumor‐supportive TANs.^[^ ^185^, ^216^, ^217^ ^]^ In addition to fostering an immunosuppressive TME, CAFs hinder T cell activation by disrupting DC maturation and antigen presentation, recruiting normal DCs, and driving their differentiation into regulatory or dysfunctional subsets.^[^ ^218^ ^]^ CAF‐derived factors, including TGF‐β, impede DC migration and antigen transport to the draining lymphatic system, while VEGF enhances immune tolerance by upregulating PD‐L1 expression on DCs.^[^ ^219^, ^220^ ^]^ Given their broad immunosuppressive functions (Figure 5), depleting CAFs has been shown to facilitate immunological control of tumor growth.^[^ ^19^, ^212^ ^]^

Advancements in single‐cell technologies have provided deeper insights into CAF heterogeneity.^[^ ^221^ ^]^ Bartoschek et al. detected spatially distinct CAF subpopulations in breast cancer that demonstrate different prognostic values and origins.^[^ ^222^ ^]^ Similarly, Cords et al. identified heterogeneous CAF phenotypes with distinct spatial distributions, marking them as independent prognostic factors in NSCLC.^[^ ^223^ ^]^ Kieffer et al. identified CAF clusters in breast cancer associated with immunosuppression and resistance to immunotherapy, revealing that myofibroblasts, characterized by ECM proteins and TGF‐β signaling, are linked to primary resistance to ICI therapies.^[^ ^224^ ^]^ Dominguez et al. profiled CAFs in pancreatic cancer during in vivo tumor evolution, revealing that stromal transition to *LRRC15* ^+^ myofibroblasts is a key determinant of poor response to anti‐PD‐L1 therapy.^[^ ^225^ ^]^ Similarly, Krishnamurthy et al. showed that TGF‐β‐dependent *LRRC15* ^+^ CAFs set the tumor‐fibroblast equilibrium to promote tumor growth, while also directly suppressing CD8^+^ T cell function and limiting ICI responses.^[^ ^226^ ^]^ Notably, certain CAF subsets can function as modulators of CD4^+^ T cells. For instance, Elyada et al. identified a CAF population that expresses MHC class II and CD74 but lacks costimulatory molecules, termed “antigen‐presenting CAFs (apCAFs),” which activate CD4^+^ T cells in an antigen‐specific manner.^[^ ^208^ ^]^ Similarly, Huang et al. demonstrated that apCAFs, derived from mesothelial cells by lineage tracing, directly induce naïve CD4^+^ T cells to differentiate into Tregs in an antigen‐specific manner in pancreatic cancer.^[^ ^227^ ^]^ Given the substantial heterogeneity and functional diversity of fibroblasts,^[^ ^228^, ^229^, ^230^, ^231^ ^]^ further comprehensive analyses are essential to fully understand the impact of CAFs on anti‐tumor immunity and ICI responses.

## 11 Spatial Architecture Associated with Clinical Outcomes and ICI Responses

The TME is not only an aggregate of diverse cellular components but also a highly organized ecosystem, where spatial architecture, defined by the arrangement of cellular and tissue components, plays a pivotal role in shaping tumor progression and clinical outcomes.^[^ ^232^ ^]^ Luca et al. defined fundamental units of cellular organization in human carcinomas and proposed a framework for large‐scale profiling of tissue cellular ecosystems, revealing conserved multicellular communities, such as myeloid‐ and stromal‐rich types, linked to poor survival.^[^ ^233^ ^]^ Ali et al. showed that tumor ecosystems, including cellular neighborhoods and cell‐cell interactions, are linked to prognosis and correlate with genomic subtypes in breast cancer.^[^ ^234^ ^]^ Additionally, Jackson et al. used spatially resolved single‐cell analyses to define the phenotypes, organization, and heterogeneity of tumor and stromal cells in breast cancer, revealing key multicellular features of the TME and identifying novel subgroups linked to distinct clinical outcomes.^[^ ^235^ ^]^ Similarly, Wu et al. used single‐cell and spatial transcriptomics to reveal spatially organized stromal‐immune niches in breast cancer, identifying nine ecotypes with distinct cell compositions and clinical outcomes.^[^ ^236^ ^]^ By integrating spatial immune profiling with gene expression data, Gruosso et al. identified distinct TIME subtypes in TNBC with unique molecular and prognostic features, enabling tumor stratification and informing immunotherapy strategies.^[^ ^237^ ^]^ Likewise, Danenberg et al. identified conserved TME structures differing in vasculatures, stromal states, and immune compositions, which vary across breast cancer subtypes and are linked to immune escape and patient stratification.^[^ ^238^ ^]^ These findings highlight the complexity and disease‐specific nature of TME spatial architecture, underscoring its critical role in shaping tumor biology, patient stratification, and clinical outcomes.

The spatial organization of the TME at both cellular and tissue levels critically influences anti‐tumor immunity and response to immunotherapy. Sorin et al. spatially profiled the tumor‐immune landscape across five histological patterns of lung adenocarcinoma (LUAD), revealing immune lineage distributions and activation states linked to distinct clinical outcomes.^[^ ^239^ ^]^ Keren et al. demonstrated that TNBC exhibits specialized multicellular architectures at tumor borders that modulate immune activity and correlate with clinical outcomes.^[^ ^240^ ^]^ Additionally, Hammerl et al. analyzed the spatial immune cell contextures in TNBC and reported that the excluded, ignored, and inflamed phenotypes exhibit distinct features associated with different clinical outcomes and mechanisms of T cell evasion.^[^ ^241^ ^]^ Enfield et al. defined four histology‐independent TME archetypes in early‐stage lung cancer, showing that T cell‐macrophage niches expand with clonal neoantigens in immune‐hot LUAD, whereas plasma and B cell niches predominate in immune‐excluded squamous cell carcinoma (LUSC).^[^ ^242^ ^]^ Notably, Liu et al. identified spatially distinct TCR repertoires in human lymphoid germinal centers and observed heterogeneous immune responses in RCC and melanoma, with T cell states and infiltration levels varying both intra‐ and inter‐clonally.^[^ ^243^ ^]^ Joshi et al. reported that the spatial heterogeneity of the TCR repertoire mirrors the mutational landscape in lung cancer, highlighting the importance of T cell distribution within the TME for effective immune responses.^[^ ^244^ ^]^ Regarding the association between spatial organization and response to ICIs, Berry et al. used multispectral imaging to identify features in pre‐treatment melanoma specimens, such as CD163^+^ PD‐L1^−^ myeloid cells and CD8^+^ FoxP3^+^ PD‐1^low/mid^ T cells, that predict response to anti‐PD‐1 therapy and stratify long‐term survival after treatment.^[^ ^245^ ^]^ Chu et al. identified Tstr cells, a stress‐response T cell state marked by heat shock gene expressions, which are primarily located in lymphoid aggregates within tumor beds or edges across cancers, and increase particularly in non‐responsive tumors after ICIs, suggesting their roles in immunotherapy resistance.^[^ ^246^ ^]^ Phillips et al. identified topographical differences ineffector PD‐1^+^ CD4^+^ T cells, tumor cells, and Tregs between responders and non‐responders, deriving a spatial biomarker, SpatialScore, which strongly correlates with pembrolizumab response in cutaneous T cell lymphomas (CTCL).^[^ ^247^ ^]^ Tumeh et al. demonstrated that CD8^+^ T cells located at the invasive tumor margin are associated with the PD‐1/PD‐L1 immune inhibitory axis and may predict response to PD‐1 blockade in metastatic melanoma.^[^ ^67^ ^]^ Conversely, Gide et al. highlighted that the proximity of immune and cancer cells plays a crucial role in mediating response to anti‐PD‐1 therapies in patients with metastatic melanoma.^[^ ^248^ ^]^ Regarding the spatial dynamics, Wang et al. mapped the multicellular tumor ecosystem in situ, revealing that ICI treatment induces TME remodeling in TNBC, where proliferating CD8^+^ TCF1^+^ T cells frequently interact with MHCII^+^ cells, a pattern that correlates with improved ICI efficacy.^[^ ^113^ ^]^ Additionally, Shiao et al. characterized the phenotypic and spatial remodeling of the TIME in TNBC following anti‐PD‐1 and radiation therapy, identifying two types of responders, one with a pre‐existing immune response and another reprogrammed by combination therapy, while non‐responders lacked immune infiltration both before and after treatment.^[^ ^249^ ^]^ These findings highlight the critical role of TME spatial architecture in orchestrating immune regulation, underscoring the importance of both pre‐existing and treatment‐induced spatial dynamics of the TIME in shaping ICI responses.

## 12 Importance of Specific TME Architectures and Intercellular Interactions in Shaping ICI Responses

Tertiary lymphoid structures (TLSs) specifically exemplify the pivotal role of spatial immune organization in driving effective anti‐tumor responses. These ectopic lymphoid formations develop in non‐lymphoid tissues during chronic inflammation, including tumors, where they serve as functional hubs for initiating and maintaining coordinated T‐ and B‐cell‐mediated anti‐tumor immunity.^[^ ^22^, ^23^, ^250^ ^]^ Studies have shown a strong association between the presence of TLSs and favorable response to ICIs.^[^ ^250^, ^251^, ^252^ ^]^ Helmink et al. demonstrated that B cells and TLSs are linked to improved response to ICI therapies in patients with melanoma.^[^ ^24^ ^]^ Similarly, Cabrita et al. found that TLSs not only enhance the efficacy of immunotherapy but also correlate with improved survival in melanoma patients.^[^ ^147^ ^]^ Vanhersecke et al. showed that the presence of mature TLSs is associated with improved objective response rates, progression‐free survival, and overall survival, regardless of PD‐L1 expression status or CD8^+^ T cell density, in a large‐scale retrospective analysis.^[^ ^253^ ^]^ These studies establish that TLSs functionally contribute to tumor control by orchestrating local adaptive immune responses, while their presence and maturation status may serve as biomarkers, both predicting patient outcomes and monitoring therapeutic efficacy of immunomodulatory regimens.

In addition to TLSs, other structures within the TME and their associations with tumor behavior and anti‐tumor immunity have also been explored. Chen et al. identified the stem‐immunity hub, distinct from mature TLSs and enriched in CD8^+^ TCF7^+^ PD‐1^+^ T cells, CCR7^+^ LAMP3^+^ DCs, and CCL19^+^ fibroblasts, which is associated with favorable PD‐1 blockade outcomes.^[^ ^254^ ^]^ Magen et al. found that intratumoral DC‐CD4^+^ Th cell niches promote CD8^+^ T cell differentiation, with progenitor CD8^+^ T cells interacting with *CXCL13* ^+^ Th cells and mregDCs, forming cellular triads that enhance anti‐tumor responses upon PD‐1 blockade in HCC.^[^ ^255^ ^]^ Schürch et al. identified distinct, conserved cellular neighborhoods (CNs) within the CRC TME, revealing that the presence of CD4^+^ PD‐1^+^ T cells within a granulocyte CN is positively correlated with survival, whereas the coupling of tumor and immune CNs, fragmentation of T cell and macrophage CNs, and disruption of inter‐CN communication are linked to poor clinical outcomes.^[^ ^256^ ^]^ Furthermore, Pelka et al. uncovered spatially organized multicellular immune hubs in CRC, identifying a myeloid cell‐attracting hub at the tumor‐luminal interface associated with tissue damage, and an MMR‐deficient‐enriched immune hub characterized by activated T cells, malignant cells, and myeloid cells expressing T cell‐attracting chemokines.^[^ ^21^ ^]^

The spatial localization of stromal components, particularly CAFs, relative to cancer and immune cells also plays a critical role in shaping anti‐tumor immunity and influencing response to ICIs. Khaliq et al. identified a conserved spectrum of spatial ecotypes in PDAC samples characterized by fibrotic, metabolic, and immunosuppressive features across different anatomical sites.^[^ ^257^ ^]^ Moncada et al. revealed a spatial colocalization between inflammatory fibroblasts and cancer cells enriched for a stress‐response gene program in PDAC.^[^ ^258^ ^]^ Notably, Liu et al. identified four CAF subtypes with distinct spatial patterns, cell interactions, and transcriptomes, whose tissue‐specific abundance influences immune infiltration, tumor phenotypes, and patient survival.^[^ ^259^ ^]^ Ma et al. performed spatial and single‐cell transcriptomic analyses of CAFs, showing that CAF proximities influence immune cell compositions, tumor progression, and ICI responses, with matrix CAFs (mCAFs) promoting angiogenesis while inflammatory CAFs (iCAFs) fostering an immunosuppressive environment.^[^ ^260^ ^]^ Importantly, Yan et al. systematically mapped spatial interactions in NSCLC, demonstrating that specific CAF subpopulations engage in cross‐talk with cancer cells and macrophages. These interactions drive pathological collagen deposition at tumor margins, creating physical barriers that limit T cell infiltration and correlate with poor clinical outcomes and resistance to combined ICI‐chemotherapy regimens.^[^ ^261^ ^]^ Consistently, Liu et al. identified a tumor‐immune barrier in HCC, composed of *SPP1*⁺ macrophages and CAFs at the tumor edge, which are driven by hypoxia‐induced interactions that remodel the matrix and limit immune infiltration, thereby reducing anti‐PD‐1 efficacy.^[^ ^262^ ^]^ Similarly, Qi et al. showed that *FAP*⁺ fibroblasts and *SPP1*⁺ macrophages are positively correlated across CRC cohorts, and their interactions, possibly driven by chemerin, TGF‐β, and IL‐1, promote desmoplastic barriers that limit T cell infiltration.^[^ ^263^ ^]^ Concordantly, Chen et al. showed that *POSTN*⁺ CAFs colocalize with *SPP1*⁺ macrophages in NSCLC, correlating with T cell exhaustion and reduced T cell infiltration.^[^ ^264^ ^]^ Peyraude et al. showed that two CAF subsets drive primary resistance to ICIs in mature TLS (mTLS)‐positive NSCLC by promoting immune exclusion, CD8⁺ T cell exhaustion, and regulatory CD4⁺ T cell infiltration, highlighting their immunosuppressive roles and potential as therapeutic targets.^[^ ^265^ ^]^

Notably, Kuett et al. performed 3D modeling within the TME, revealing cellular and microenvironmental heterogeneity, as well as cell‐level tissue organization, which are not captured by traditional 2D analyses, highlighting the need to study the TME in three dimensions.^[^ ^266^ ^]^ Lin et al. further demonstrated that at the tumor invasive margin, where tumor, normal, and immune cells converge, T cell suppression is orchestrated by multiple cell types. Using 3D imaging, they showed that features such as TLSs, which appear discrete in 2D, are often interconnected and exhibit continuous molecular gradients.^[^ ^267^ ^]^ As spatial profiling technologies advance, capturing the complexity of these cellular architectures to build a holistic view of the intricate molecular mechanisms shaping tumor ecosystem dynamics and ICI responsiveness warrants further investigation.

## 13 Role of Microbiota and Metabolites in Immune Reprogramming and ICI Responses

The gut microbiome, which consists of bacteria, fungi, viruses, archaea, and protists, plays a crucial role in human health and disease progression, including cancer.^[^ ^268^, ^269^, ^270^ ^]^ Beyond its impact on overall health, the gut microbiota significantly influences immune responses, both locally within the gut and systemically, thus affecting the immune surveillance and efficacy of cancer therapies, including ICIs.^[^ ^271^, ^272^, ^273^ ^]^ Schluter et al. demonstrated that the gut microbiota has a profound effect on immune cell dynamics in cancer patients undergoing hematopoietic cell transplantation, revealing important, time‐dependent interactions between microbial composition and immune function.^[^ ^274^ ^]^ Additionally, Iida et al. showed that a healthy and intact commensal microbiota is essential for optimal therapeutic responses, likely functioning by modulating myeloid cells within the TME and enhancing anti‐tumor immunity.^[^ ^275^ ^]^ Viaud et al. further emphasized that the gut microbiota influences anti‐tumor immune responses by promoting the generation of pathogenic Th17 (pTh17) cells and memory Th1 responses, both of which are critical for effective immune surveillance and anti‐tumor immunity.^[^ ^276^ ^]^

**Figure 6:**
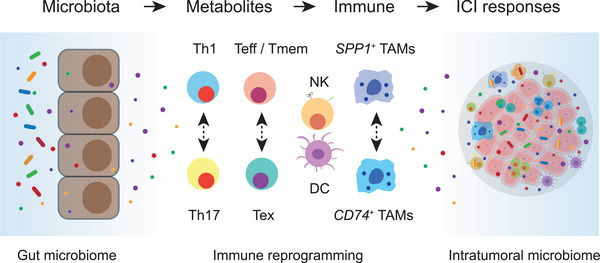
The gut and intratumoral microbiomes and their immune regulation via metabolites. Compositions of both gut and intratumoral microbiota, along with their derived metabolites, significantly influence immune cell reprogramming. Studies have demonstrated that microbiota‐derived metabolites can modulate the balance between Th1 and Th17 cells, regulate the dynamics between Tex and Teff / Tmem cells, influence the phenotypic polarization of TAMs, and affect the interactions between DCs and NK cells.

Emerging evidence highlights the crucial role of microbiota in shaping ICI responses (**Figure** **6**). Routy et al. demonstrated that the gut microbiome functions in modulating the efficacy of PD‐1‐based immunotherapy against epithelial tumors.^[^ ^276^ ^]^ Chaput et al. found that a baseline gut microbiota enriched with *Faecalibacterium* and other *Firmicutes* is associated with better clinical responses to ipilimumab, an anti‐CTLA‐4 therapy, and a higher incidence of ipilimumab‐induced colitis, suggesting that a microbiota enriched in specific bacterial species may influence both therapeutic outcomes and side effects.^[^ ^277^ ^]^ Gopalakrishnan et al. identified significant microbiome differences between melanoma patients responding and not responding to anti‐PD‐1 therapy, reporting that fecal microbiota transplantation (FMT) from responders enhances immune responses in germ‐free mice.^[^ ^278^ ^]^ Additionally, Cao et al. demonstrated that the gut microbiota boosts ICI efficacy by increasing the frequency of CD8⁺ T cells, CD4⁺ T cells, and γδ T cells, reducing glycolysis, and converting CD8⁺ Tex into Teff / Tmem cells. Moreover, the microbiota reprograms Spp1⁺ TAMs into Cd74⁺ antigen‐presenting cell (APC)‐like TAMs, an effect associated with therapeutic outcomes during FMT.^[^ ^279^ ^]^ Consistently, Lam et al. revealed that microbiota‐derived STING agonists induce type I interferon (IFN‐I) production in intratumoral monocytes, which enhances macrophage polarization and promotes NK‐DC crosstalk, significantly improving ICI responses.^[^ ^280^ ^]^

The precise mechanisms by which the microbiome modulates ICI responses remain incompletely understood. Recent studies have highlighted that metabolites produced by microbes are sensed by immune cells, influencing the balance between pro‐inflammatory and anti‐inflammatory responses.^[^ ^281^, ^282^, ^283^ ^]^ Arpaia et al. demonstrated that intestinal microbes not only provide essential nutrients but also protect against infections, influencing immune balance through bacterial metabolites that regulate the immune system's inflammatory responses.^[^ ^281^ ^]^ Mager et al. identified *B. pseudolongum*, a key species of microbiota, as a producer of inosine, which enhances ICI responses by activating anti‐tumor T cells through the translocation of inosine.^[^ ^284^ ^]^ Jia et al. found that the commensal bacterium *Lactobacillus johnsonii* correlates with improved ICI responsiveness, and its metabolite, indole‐3‐propionic acid (IPA), enhances immunotherapy efficacy by modulating T cell stemness and promoting CD8⁺ Tpex generation through increased H3K27 acetylation at the Tcf7 super‐enhancer region.^[^ ^285^ ^]^ Furthermore, Wang et al. discovered that the microbial metabolite trimethylamine N‐oxide (TMAO) enhances anti‐tumor immunity in TNBC, suggesting that microbiota‐metabolite‐immune interactions could be exploited as a novel therapeutic strategy to boost immunotherapy efficacy.^[^ ^286^ ^]^ Similarly, Mirji et al. showed that TMAO administration reduces tumor growth, promotes an immuno‐stimulatory TAM phenotype, and activates Teff cell responses in PDAC‐bearing mice.^[^ ^287^ ^]^ Furthermore, short‐chain fatty acids (SCFAs), produced by gut microbiota, are also essential in modulating immune responses and are associated with clinical responses to ICIs.^[^ ^278^, ^283^, ^288^, ^289^ ^]^ Nomura et al. demonstrated that SCFAs are linked to clinical responses to nivolumab or pembrolizumab in solid tumor patients, highlighting the importance of microbiota‐derived metabolites in regulating ICI efficacy.^[^ ^290^ ^]^ Bachem et al. found that the SCFA butyrate enhances cellular metabolism, bolsters memory potential in CD8⁺ T cells, and is necessary for optimal recall responses upon antigen re‐encounter, thus promoting long‐term survival and memory formation in CD8⁺ T cells.^[^ ^291^ ^]^ On the other hand, Pinato et al. showed that prior antibiotic treatment negatively affects cancer patients' response to ICIs, emphasizing the need for a balanced gut microbiome for optimal ICI outcomes^[^ ^292^ ^]^

Recent advances have solidified that modulating the microbiome holds substantial potential for overcoming resistance to ICIs and improving treatment safety.^[^ ^271^, ^273^, ^293^, ^294^ ^]^ Matson et al. found that the composition of the commensal microbiota correlates with clinical response, with *Bifidobacterium longum*, *Collinsella aerofaciens*, and *Enterococcus faecium* enriched in responders. Fecal transfer from these patients improvesd tumor control and anti‐PD‐L1 efficacy.^[^ ^289^ ^]^ Sivan et al. demonstrated that *Bifidobacterium* are linked to anti‐tumor effects, and their oral administration mimick the effects of PD‐L1 therapy, suggesting that microbiota manipulation could significantly enhance cancer immunotherapy efficacy.^[^ ^295^ ^]^ Baruch et al. showed that FMT in anti‐PD‐1‐refractory patients with metastatic melanoma leads to clinical responses, accompanied by favorable changes in immune cell infiltrates and gene expression profiles in both the gut lamina propria and the TME.^[^ ^296^ ^]^ Similarly, Davar et al. reported that FMT combined with anti‐PD‐1 therapy benefits PD‐1‐refractory melanoma patients by inducing lasting microbiota changes, increasing CD8⁺ T cell activation, and reducing IL‐8⁺ myeloid cells, accompanied by distinct proteomic and metabolomic signatures.^[^ ^297^ ^]^ Dizman et al. demonstrated that CBM588, a bifidogenic live bacterial product, may enhance the clinical outcomes of metastatic RCC patients treated with nivolumab‐ipilimumab by modulating the gut microbiome and boosting the ICI response.^[^ ^298^ ^]^ Consistently, Vétizou et al. showed that the antitumor effects of CTLA‐4 blockade depend on specific *Bacteroides* species, further supporting the role of the microbiome in modulating ICI efficacy.^[^ ^299^ ^]^

Dietary interventions have also been shown to significantly alter the human gut microbiome, influencing immune function and cancer therapy responses.^[^ ^300^, ^301^, ^302^, ^303^ ^]^ Studies show that dietary fiber rapidly and reproducibly alters the gut microbiome, correlating with improvements in glucose metabolism and specific microbiota composition changes that enhance immune function.^[^ ^304^, ^305^, ^306^ ^]^ Spencer et al. found that dietary fiber and probiotics positively influence the gut microbiome and melanoma immunotherapy responses, suggesting that diet plays a vital role in modulating immune reprogramming through the microbiome.^[^ ^302^ ^]^ However, the microbiome can also exert pro‐tumor effects. Wu et al. reported that a human colonic commensal promotes colon tumorigenesis through the activation of Th17 cell responses.^[^ ^307^ ^]^ Bullman et al. showed that *Fusobacterium*, along with *Bacteroides*, *Selenomonas*, and *Prevotella*, persists in both primary and metastatic colorectal tumors, highlighting microbiome stability and suggesting antimicrobial interventions as a potential treatment for *Fusobacterium*‐associated CRC.^[^ ^308^ ^]^

In addition to the gut microbiome, tumor‐associated microbiota, an integral component of the TME, also plays a pivotal role in tumor progression and immune modulation. Nejman et al. conducted a large‐scale analysis of 1,526 tumors and adjacent normal tissues across seven cancer types, uncovering tumor type‐specific microbiome profiles. They found that most intratumoral bacteria are intracellular, residing within both cancer and immune cells. Notably, microbial compositions and predicted functions correlate with tumor type, molecular subtype, smoking status, and response to immunotherapy.^[^ ^309^ ^]^ Galeano Niño et al. showed that bacterial communities colonize specific microniches within tumors, which are characterized by low vascularization and a highly immunosuppressive environment. In addition, bacterial‐rich regions are associated with malignant cells that exhibit lower Ki‐67 expression compared to bacteria‐negative tumor areas, underscoring the potential impact of the microbiota on tumor biology.^[^ ^310^ ^]^ Bender et al. reported that metabolites derived from dietary tryptophan, released by intratumoral *Lactobacillus reuteri*, can enhance the efficacy of ICI therapy.^[^ ^311^ ^]^ Similarly, Riquelme et al. found that long‐term survivors of PDAC exhibit greater alpha‐diversity in their tumor microbiome. They also identified a distinct microbial signature, comprising *Pseudoxanthomonas*, *Streptomyces*, *Saccharopolyspora*, and *Bacillus clausii*, which is predictive of survival outcomes.^[^ ^312^ ^]^ These findings suggest that the tumor microbiome, interacting with the gut microbiome, plays a pivotal role in shaping immune responses and influencing disease progression.

In brief, the microbiota, through its compositions and metabolites, plays an important role in shaping the immune landscape and modulating ICI responses. The dynamic interactions between microbiota, immune cells, and cancer cells offer a promising avenue for improving cancer immunotherapy. By modulating the microbiome, either through dietary modifications, probiotics, or FMT, we may be able to overcome resistance to ICIs and enhance their efficacy, ultimately leading to more effective and personalized cancer treatment.

## 14 Conclusion and Outlook

Immunotherapy has revolutionized cancer treatment by harnessing the immune system to target tumors; however, not all patients respond to ICIs, and the TME plays a critical role in this variability.^[^ ^5^, ^313^ ^]^ Although cellular components of the TME have been well‐characterized, their dynamic changes and impacts on ICI responses are not yet fully understood. In this review, we summarize immune cell dynamics in the TME, peripheral blood, and TDLNs, with a focus on T cells that are central effector cells responsible for cancer cell elimination and major targets of ICIs. CD8^+^ T cells, especially those with tissue‐resident or exhausted phenotypes, have emerged as strong predictors of favorable ICI responses, while those with central memory phenotypes or expressing TCF1/TCF7 play pivotal roles in sustaining anti‐tumor immunity post‐treatment. CD4^+^ T cells are increasingly recognized as crucial mediators of anti‐tumor immunity, yet their functional diversity remains incompletely characterized, though emerging evidence highlights their complex crosstalk with B cells as a critical determinant of ICI efficacy.^[^ ^121^, ^151^ ^]^ We also offer a comprehensive review of additional immune cell components, including macrophages, DCs, B cells, and neutrophils, focusing on their intricate interactions with T cells within the TME. By examining these cellular interactions, we highlight their critical contributions to the efficacy of ICIs and provide insights into how they can be leveraged to enhance immunotherapy outcomes.

We also present a detailed review of the role of stromal components, particularly CAFs, in modulating response to ICIs. CAFs are pivotal in shaping the TME.^[^ ^226^, ^229^, ^261^ ^]^ By regulating immune cell infiltration, cytokine secretion, and ECM remodeling, CAFs contribute to immune evasion and tumor progression, thereby significantly affecting ICI efficacy.^[^ ^206^, ^207^, ^208^ ^]^ Importantly, the spatial distribution of CAFs critically shapes immune cell accessibility, antigen presentation, and immune escape, underscoring the importance of spatial architecture in the TME. We thus exemplify the spatial organization of cellular components within the TME, which is crucial in dictating anti‐tumor immune responses and determining the efficacy of ICIs.^[^ ^21^, ^113^, ^261^ ^]^ With the advent of spatial transcriptomics, proteomics, and advanced imaging technologies, a deeper understanding of the spatial architecture of the TME will further unravel the organizations and interactions between cancer cells and stromal cells within their native context, which will facilitate the development of novel therapeutic targets and biomarkers for predicting ICI efficacy.

The field of immunotherapy continues to evolve, with the microbiome emerging as another critical factor in shaping immune responses and influencing ICI therapies.^[^ ^314^, ^315^ ^]^ Specific microbiota profiles correlate with better clinical outcomes in patients treated with ICIs,^[^ ^278^, ^295^, ^299^ ^]^ underscoring the importance of gut health in immune modulation. Future research will likely focus on deciphering the mechanisms by which the microbiome impacts immune responses and exploring strategies to manipulate the microbiota, such as through probiotics or FMT, to enhance treatment outcomes. In addition, metabolic reprogramming has also been shown to critically impact anti‐tumor immunity.^[^ ^316^, ^317^ ^]^ Cancer cells, immune cells, and stromal components undergo considerable metabolic reprogramming within the TME, which profoundly affects immune cell function and tumor progression.^[^ ^318^, ^319^, ^320^, ^321^, ^322^ ^]^ Metabolic pathways, such as glycolysis, oxidative phosphorylation, and fatty acid metabolism, influence T cell activation, exhaustion, and differentiation.^[^ ^323^, ^324^, ^325^, ^326^, ^327^, ^328^ ^]^ Understanding how metabolic shifts within the TME influence the immune response and ICI therapies will be critical for optimizing immunotherapy. Targeting key metabolic pathways, such as IDO, mTOR, and AMPK, could provide new therapeutic avenues to overcome immune suppression and enhance anti‐tumor immunity.

Improving the response rate to ICIs remains a major challenge in cancer immunotherapy. The use of dual or multiple ICIs targeting distinct immune checkpoints has shown promising potential to overcome resistance mechanisms and insufficient immune activation. For example, the combination of PD‐1/PD‐L1 and CTLA‐4 inhibitors has demonstrated enhanced efficacy across multiple cancer types.^[^ ^329^, ^330^, ^331^, ^332^ ^]^ Studies have shown that anti‐CTLA‐4 and anti‐PD‐1 checkpoint blockades target distinct subsets of TILs,^[^ ^333^, ^334^ ^]^ and their synergistic effects lead to increased T cell infiltration and sustained anti‐tumor immune responses. However, this approach is also associated with a higher risk of immune‐related adverse events (irAEs),^[^ ^331^, ^335^ ^]^ necessitating careful patient selection and management. Other immune checkpoints, such as LAG‐3, TIM‐3, and TIGIT, are also being explored in combination with PD‐1/PD‐L1 inhibitors.^[^ ^336^, ^337^ ^]^ These emerging targets modulate complementary pathways of immune suppression and may provide synergistic benefits when combined with conventional ICIs. Combining multiple checkpoint inhibitors may lead to more durable responses, particularly in tumors with an immunosuppressive microenvironment.

Combining ICIs with other therapies, such as chemotherapy, is emerging as another promising strategy to overcome resistance and enhance the efficacy of ICIs. Chemotherapeutic agents like doxorubicin and oxaliplatin could induce immunogenic cell death (ICD), releasing tumor antigens that stimulate anti‐tumor immune responses.^[^ ^338^, ^339^, ^340^, ^341^ ^]^ Chemotherapy can also reverse immune suppression in the TME and increase immune cell infiltration, particularly cytotoxic T cells, thus improving ICI efficacy.^[^ ^342^, ^343^, ^344^ ^]^ Combining chemotherapy with ICIs has shown promise in cancers like NSCLC and TNBC.^[^ ^345^, ^346^, ^347^, ^348^ ^]^ However, combining different chemotherapeutic agents with ICIs can yield varying outcomes. For instance, in TNBC, the combination of nab‐paclitaxel with the anti‐PD‐L1 antibody atezolizumab has demonstrated superior clinical responses compared to the combination of paclitaxel with atezolizumab.^[^ ^96^, ^347^, ^349^, ^350^ ^]^ In addition, responses to these combination therapies may vary depending on tumor‐specific molecular and immune profiles.^[^ ^96^, ^350^, ^351^ ^]^ Therefore, further research is essential to optimize chemotherapeutic agents and schedules, manage adverse events, and identify predictive biomarkers to guide personalized treatment strategies.

In summary, this review consolidates current insights into the roles of immune cells and stromal components within the TME, with a particular focus on their impact on ICI responses. It highlights the significance of CD8^+^ T cell subsets, the emerging roles of CD4^+^ T cells and B cells, the influence of neutrophils and CAFs, and the importance of spatial structures in immune modulation. These summaries provide a deeper understanding of tumor‐immune interactions and offer promising avenues for enhancing the effectiveness of immunotherapy.

## Conflict of Interest

The authors declare no conflict of interest.

## Author Contributions

Y.Z. and Z.L. supervised and conceptualized the study; Y.Z. wrote the original draft; Y.Z. and Z.L. performed the review and editing; Z.L. and Y.Z. acquired the funding.
